# Determinants of the decision to enroll in community-based health insurance among households in the West Guji Zone, Oromia State, southern Ethiopia, in 2022

**DOI:** 10.3389/frhs.2025.1559578

**Published:** 2025-05-15

**Authors:** Gelgelo Wodessa, Miesa Gelchu, Anteneh Fikrie, Gemechis Tuke

**Affiliations:** School of Public Health, Institute of Health, Bule Hora University, Bule Hora, Ethiopia

**Keywords:** community-based health insurance, decision, determinants, enrolment, West Guji, southern Ethiopia

## Abstract

**Background:**

In recent years, the Ethiopian government has introduced community-based health insurance (CBHI) schemes to increase healthcare accessibility and affordability. Despite these efforts, enrolment rates remain low, posing challenges to achieving universal health coverage. This study investigates the determinants influencing household enrolment decisions in community-based health insurance within the West Guji Zone, Oromia Regional State, southern Ethiopia, in 2022.

**Methods and materials:**

A community-based, unmatched case-control study was conducted among 690 randomly selected households (345 cases and 345 controls) in the West Guji Zone from 15 April to 15 June 2022. Data were collected via a pretested and structured face-to-face interviewer-administered questionnaire. The data were entered into EpiData 3.1 and analyzed via SPSS Version 26. Bivariate and multivariate binary logistic regression models were used to identify the determinants of enrolment decisions in community-based health insurance. An adjusted odds ratio (AOR) with a 95% confidence interval and *p*-value <0.05 was used to declare statistically significant associations.

**Results:**

The findings of this study revealed that factors such as no formal education (AOR = 2.885, 95% CI: 1.252, 6.648), negative perception (AOR = 0.067, 95% CI: 0.040, 0.112), poor and middle wealth index (AOR = 0.307, 95% CI: 0.166, 0.569), community solidarity practices (AOR = 4.266, 95% CI: 2.352, 7.736), trust in the CBHI scheme (AOR = 4.782, 95% CI: 2.926, 7.816), quality of service (AOR = 2.209, 95% CI: 1.324, 3.687), availability of prescribed drugs (AOR = 1.829, 95% CI: 1.102, 3.035), and satisfaction with services (AOR = 3.209, 95% CI: 1.937, 5.315) were identified as significant determinants of CBHI enrolment decisions.

**Conclusion:**

This study revealed that a lack of formal education, negative perceptions, and a lower wealth index negatively impact CBHI enrolment. However, community solidarity practices, trust, quality of service, availability of prescribed drugs, and satisfaction positively influence CBHI enrolment. This study highlights the need for targeted interventions to increase community awareness, foster community solidarity at the local level, increase trust in the health system, and increase the affordability of premiums, thereby promoting community-based health insurance enrolment and achieving universal health coverage in Ethiopia.

## 1 Introduction

It is rightly said that health is wealth (1). An individual's and society's health are severely impacted by excess out-of-pocket (OOP) payments, changing lifestyle patterns, and the unaffordability of medical-related services (2). This leads to a variety of illnesses and diseases, and since the expense of treating these illnesses is increasing quickly, it is vital to invest in community-based health insurance (CBHI) to protect the local community from excessive medical expenses (3).

Globally, there is an enormous mismatch between countries’ health financing needs and health spending (4). Approximately 11.7% of the world's population faces catastrophic health expenditure, while 25 million households are impoverished because of direct healthcare payments (5). Every year, more than 150 million individuals in Africa face high healthcare costs, with the majority of these treatment seekers living in poverty as a result of out-of-pocket payments (6).

In addition, low- and middle-income countries (LMICs) have developed alternative health financing strategies for out-of-pocket expenses to protect their population from catastrophic health service expenditure (7, 8). More importantly, as a community health financing mechanism, CBHI seems more appropriate for LMICs where governments have limited fiscal space and overreliance on OOP spending (9). Over 90% of healthcare financial difficulties and their consequences have occurred in sub-Saharan African countries, where resources are limited (10).

CBHI has emerged as a context-appropriate risk-pooling mechanism to provide some financial protection to populations without access to formal health insurance and as an emerging strategy for providing financial protection against health-related poverty (11). The CBHI decouples the time of payment from the time of use of services, which is relevant for rural households because of seasonal variations in their incomes and provides access to healthcare with financial risk protection (12).

Henceforth, CBHI has gained popularity as a makeshift health financing mechanism for OOP payments in poor communities by combining resources and risk at the individual and community levels (13).

Ethiopia, a sub-Saharan African country, has many difficulties in delivering affordable healthcare to its citizens, as people lack access to needed medical services, medications, and pharmaceutical supplies (13). One national report in Ethiopia showed that only 48% of households are enrolled in pilot schemes, with large variations within and between districts. This percentage ranges from as low as 25% in Deder and as high as 100% (universal enrolment) in Yirgalem (14, 15).

The Ethiopian government set goals for 2020 to achieve CBHI schemes, aiming to have 80% of districts and 80% of households enrolled to reduce OOP health expenditure to less than 15% and increase general government expenditure on health (GGE) as a share of total GGE from 6% to 10%; nevertheless, these ambitious targets were not attained (16). In addition, demonstrating the existing regional variation, the 2019 Ethiopian Mini Demographic and Health Survey (EMDHS) results suggest that 28% of households were enrolled in community-based health insurance, rural households (32%) were more likely to be enrolled than urban households (19%), and at the population level, three out of 10 Ethiopians (28%) were enrolled, whereas 72% were not (17). In general, many studies suggest that the magnitude of enrolment in this country is low (18).

In recent years, the Ethiopian government has made efforts to improve healthcare accessibility and affordability through the implementation of CBHI schemes (19). However, the uptake and enrolment rates in these schemes remain low, hindering the achievement of universal health coverage (20).

Despite the low enrolment rate, little is known about the factors that influence CBHI enrollment decisions. In addition, the available data on this limited coverage have identified the factors that discourage the enrolment of households (21). A review of the literature also revealed a methodological gap; to our knowledge, no similar study has been conducted in the study area in the West Guji Zone in Ethiopia. The objective of this study was to assess the determinants of the decision to enroll in community-based health insurance in households in the West Guji Zone, southern Ethiopia, in 2022.

## 2 Methods and materials

### 2.1 Study setting

The study was conducted in the West Guji Zone of Oromia, southern Ethiopia. It is 467 km from Addis Ababa on the paved Addis Ababa-Moyale route. The zone is administratively composed of nine districts. The total number of eligible households for CBHI was estimated to be 219,017, and the number of those enrolled in CBHI schemes was only 23,745, according to the West Guji Zone Health Office report of 30 March 2022. The health facilities in the zone include approximately 196 health posts, 42 health centers, three primary hospitals, and one comprehensive specialized hospital.

### 2.2 Study design and period

The community-based unmatched case-control study design was conducted from 15 April to 15 June 2022.

### 2.3 Source and study population

All households in the West Guji Zone were the source population, whereas randomly selected households in the selected kebeles (i.e., the smallest administrative unit in Ethiopia) in the West Guji Zone during the data collection period composed the study population. Households that registered and renewed their membership in the CBHI for 2022 and households not registered for the CBHI were included in the study as cases and controls, respectively. Those for which the government covers their CBHI enrolment payment and critical illnesses were excluded from the study.

### 2.4 Sample size determination and sampling procedure

The double population proportion formula using Epi Info version 7.0.8.3 was used to determine the sample size via the following assumption: at the 95% confidence level, a power of 80% was used. The ratio of controls to cases (r) was 1, adjusted odds ratio (AOR) = 2.2, the percentage of controls exposed (P1) was 17.2, and the percentage of cases exposed (P2) was 29.5% (22). Considering the design effect (DEFF = 2) and the possible non-response rate of 10%, a sample size of 690 (345 cases and 345 controls) households was included in the study.

The study participants were drawn via a multistage sampling technique. In the first stage, three districts were chosen at random (lottery method) from among the nine districts (accounting for 30% of the districts located in the West Guji Zone). In the second stage, 21 kebeles were selected at random (lottery method) from 65 kebeles. A list of households to be enrolled as cases from the CBHI membership register and a list of households to be enrolled as controls were subsequently obtained from each kebele's administration household record list. The sample size was then proportionally allocated for selected kebeles on the basis of the number of households enrolled and not enrolled in the CBHI in each kebele. Finally, the cases and controls were selected via systematic sampling techniques.

### 2.5 Data collection procedures and quality assurance

Data collection tools were adapted by reviewing the literature and manuals from prior studies (22–24). The questionnaire was prepared in English, then translated to the regional language “Afaan Oromo” by fluent speakers of both languages, and then translated back to English to maintain the consistency of the questionnaire. Four BSc-holding nurses and two BSc-holding public health officers participated as the data collectors and supervisors, respectively. The data collectors used pretested, structured, face-to-face interviewer-administered questionnaires.

The supervisors oversaw and guided the data collectors during the data collection process by checking the completeness of the required data and correcting errors in the field.

To maintain the quality of the data, a pretest was conducted on 5% of the sample size in other kebeles in the Galana District in the source population. Training was given to the data collectors and supervisors over 2 days. Before the data collection, the consistency, coherence, and time to complete the questionnaire to be checked.

### 2.6 Data processing and analysis

The data were entered into EpiData 3.1, and SPSS version 26 was used for the analysis. Inconsistent and missing values were checked in the data. Descriptive statistics were used to characterize the study's aims using relevant variables. Bivariate and multivariate binary logistic regression models were used to determine the determinants of enrolment in the CBHI.

Variables with a *p*-value of ≤0.25 in the bivariable logistic regression analysis were entered and further computed in a multivariate binary logistic regression model to control for confounding variables. The assumptions of model fitness and multicollinearity between independent variables were assessed via the Hosmer–Lemeshow test statistic and a variance inflation factor <10, respectively. The reliability analysis was conducted via Cronbach's alpha, which was found to be 0.86. This indicates a high level of internal consistency among the items, suggesting that the measurement tool reliably reflects the construct being evaluated. Odds ratios (ORs) with 95% CIs and *p*-values <0.05 were considered statistically significant determinants.

### 2.7 Study variables and operational definitions

The dependent variable for the study was the enrolment decision in the CBHI, and the explanatory variables are as follows:

*Sociodemographic and economic characteristics of the household*: age, sex, religion, ethnicity, marital status, educational status, occupational status, family size, source of income, and wealth index.
*Scheme-related factors*: having information about CBHI, time waiting for a CBHI service card after paying, year-based payment without services, high payment compared with OOP, convenience of premium collection time, accidental change in rules, no trust in scheme management, and inappropriate use of service cards.
*Medical/health-related factors*: waiting time, poor service in governmental institutions, chronic illness in the family, satisfaction with service in the nearby facility, and drug availability.
*Cases*: households that registered with community-based health insurance and renewed their membership in 2022.
*Controls*: households that were not registered with community-based health insurance until 2022.
*Knowledge of the CBHI*: The participants’ knowledge of CBHI was assessed via five questions that included the concepts, roles, and beneficiaries of the CBHI. The participants who answered these questions correctly were categorized as having a correct response, whereas those who answered incorrectly were classified as having an incorrect response. Participants who responded “I do not know” were also included in a separate category. Each question carried equal weight, with a score of 1 assigned to each correct response, a score of 2 for each incorrect response, and a score of 0 for “I do not know” responses. Consequently, the aggregate score for all the knowledge questions ranged from 0 to 5 points. Participants’ overall knowledge was considered “good” if they scored 4 or 5 (representing ≥70% of the points) and otherwise labeled “poor” (18).
*Perception of study participants toward the CBHI scheme*: The perception of the study participants toward the CBHI scheme refers to an individual's or household's beliefs, opinions, and views regarding the CBHI scheme's effectiveness in providing financial protection against healthcare costs (6). Participants were asked to express their opinions in four perception tools via a five-point Likert scale ranging from “1 strongly disagree” to “5 strongly agree.” To rank the tools on the basis of relative importance, the minimum attainable score was first determined. The maximum attainable score for each grade in the scale was subsequently calculated by multiplying the minimum attainable score (4) by the corresponding grades (1, 2, 3, 4, and 5). Finally, respondents with scores greater than the median were classified as having positive perceptions, whereas those with scores lower than the median were categorized as having negative perceptions (22).

Equb is a rotating savings and credit association where members contribute a fixed amount of money regularly, and the collected funds are then distributed to each member in turn, providing a mechanism for savings and access to credit (25).

Edir is a traditional, informal, community-based institution that offers financial and social support, primarily serving as a form of social insurance and mutual aid, particularly for members’ families in the event of death (25).

## 3 Results

### 3.1 Sociodemographic characteristics of the study participants

In this study, a total of 345 cases and 345 controls participated, with a response rate of 100% for both groups. The mean age of the respondents for the cases was 39.7 years, with a standard deviation (SD) of ±7.859, and for the controls, it was 39.83 years, with an SD of ±7.435. With respect to the wealth index of the households, a comparable number of poor households were found among both the cases and the controls, with 127 (36.8%) and 145 (42%), respectively. In terms of education, approximately 162 (47%) of the respondents in the case group had formal education, whereas 28 (8.1%) respondents in the cases and 44 (12.8%) respondents in the controls had only completed education above the secondary level. In addition, approximately 151 (43.8%) case and 118 (34.2%) control households took less than 1 h to reach the nearby health institution. Furthermore, the majority of households practiced mixed types of agriculture (Supplementary Table S1).

### 3.2 Perception of the CBHI in households

In this study, a significantly greater proportion of cases (145, 42%) than controls (57, 16.5%) agreed with the statement that CBHI management is trustworthy. Moreover, the quality of service provided by the CBHI was reported to be satisfactory by a greater number of cases (179, 51.9%) than by the controls (73, 21.2%) (Supplementary Table S2).

### 3.3 Medical and health-related characteristics of the respondents

Concerning medical and health-related factors, comparable households had a non-communicable disease prevalence of 37.1% (128) among the cases and 42.3% (146) among the control group. With respect to the availability of prescribed essential drugs, 260 (75.4%) cases and 163 (47.2%) controls received service provision. With respect to satisfaction with the CBHI service, 260 (75%) cases and 163 (36.5%) controls reported that they were satisfied with the CBHI services (Supplementary Table S3).

### 3.4 Determinants of the decision to enroll in CBHI schemes

In the bivariable analysis, variables were found to be significant at a *p*-value <0.25 with a 95% confidence interval and were further analyzed in a multivariable binary logistic regression model to control for confounders and determine the effect of each independent variable on the likelihood of CBHI enrolment. According to the final multivariable logistic regression analysis, several factors were significantly associated with the decision to enroll in the CBHI.

Individuals with no formal education had approximately three times greater odds of enrolling in CBHI than those with above-secondary-level education (AOR = 3, 95% CI: 1.25–6.64). Household heads with a negative perception of the CBHI had 94% lower odds of enrolling than their counterparts did (AOR = 0.06, 95% CI: 0.04–0.11). Furthermore, households in the poor wealth quintile (AOR = 0.31, 95% CI: 0.16–0.56) and middle wealth quintile (AOR = 0.44, 95% CI: 0.22–0.84) were 69% and 56% less likely to enroll in the CBHI, respectively, than those in the rich wealth quintile. Compared with non-participants, respondents who participated in local solidarity practices (Equb and Edir) were four times more likely to enroll in CBHI (AOR = 4.26, 95% CI: 2.35–7.73). In addition, households that had trust in the CBHI scheme's management had nearly five times higher odds of enrolling (AOR = 4.78, 95% CI: 2.92–7.81) than did those without trust. The respondents who rated the quality of CBHI service provision as good had two times greater odds of enrolling (AOR = 2.20, 95% CI: 1.32–3.68) than those who rated it as poor. Moreover, the odds of enrolment in CBHI were nearly two times greater among household heads who had access to prescribed drugs (AOR = 1.83, 95% CI: 1.10–3.03). Finally, respondents who expressed satisfaction with CBHI services were three times more likely to enroll (AOR = 3.20, 95% CI: 1.93–5.31) than their counterparts were (Supplementary Table S4).

## 4 Discussion

The objective of this study was to identify the determinants influencing households’ enrolment decisions in the CBHI scheme among households in the West Guji Zone, southern Ethiopia. These determinants include factors such as the perception of households toward the CBHI, absence of formal education, and participation of communities belonging to poor and middle-wealth quintiles in solidarity practices, trust in the CBHI scheme, quality of service, availability of prescribed drugs, and satisfaction with the services provided by the CBHI packages.

In this study, the odds of deciding to enroll in CBHI were 93.3% lower among household heads who had a negative perception of the CBHI scheme than among their counterparts. This finding is consistent with a study conducted in the Armachiho district, North China (22); a pilot study on the evaluation of the CBHI performed nationally in Ethiopia (26); and a study performed in rural Burkina Faso (27). This similarity might be due to the methodological and socioeconomic characteristics of the community. This implies that a negative perception of CBHI blocks the uptake of the service and utilization of the service and exposes the community to OOP expenses (28).

In this study, participating in local solidarity practices (Equb or Edir) increased the odds of enrolling in the CBHI. This result is consistent with different studies performed in the Jimma Zone and West Gojjam Zone in the Sidama Region and a pilot study on the evaluation of CBHI performed nationally across Ethiopia and Senegal (29–32). The similarities are due to the study methods and sociocultural characteristics of the community, which imply that the community practices are related to solidarity practices in their settings.

This study revealed that respondents who had no formal education had greater odds of deciding to enroll in CBHI than did respondents who had above-secondary educational status. This finding is constrained by studies conducted in Gida Ayana, western Ethiopia (23); the Boricha District in the Sidama Regional State, Ethiopia (10); and the Southern Nations, Nationalities, and Peoples' Region (SNNPR) in Ethiopia (33). This implies that efforts to increase enrolment in CBHI need to address the problem of communities with lower socioeconomic status. Likewise, individuals with a higher educational status may not be eligible for CBHI because of their employability and the low quality of CBHI services.

In addition, this study revealed that households with poor and middle-wealth statuses were less likely to enroll in the CBHI scheme than those with the richest status. This result is inconsistent with different studies conducted in Ethiopia (23, 31, 32). The reason behind the inconsistency may be the difference in the study setting, methodological differences, and pressure from CBHI officials. This finding shows that CBHI membership is based on the activity of officials and that the community may consider it a valueless scheme. Moreover, CBHI membership is mandatory for the informal sector of society.

This study also reveals that the odds of deciding to enroll in the CBHI were greater among households that had trust in the scheme. This finding is in line with studies performed in different settings in Ethiopia (10, 22). This is due to the study design used and the sociodemographic and economic characteristics of the society, which implies that the management system of the CBHI program is weak, and it offers a low quality of service. Furthermore, respondents who rated the quality of CBHI schemes as good were more likely to enroll in the CBHI. This finding is consistent with studies conducted in Ethiopia (22, 34). This might be due to methodological and health infrastructure-related similarities. This indicates that there is better enrollment if full packages of health services are provided by corporations to solve the health problems of communities.

Furthermore, households that received essential drug provision were more likely to decide to enroll in CBHI. This study is in line with a study conducted in Ethiopia (34, 35). This similarity is also due to the poor health infrastructure that the health facility serves above its catchment area. This implies poor delivery of prescribed drugs, which prevents the community from enrolling in CBHI. On the other hand, respondents who were satisfied with the CBHI scheme service had higher odds of enrolling in CBHI. This result was consistent with different studies performed in Ethiopia (10, 34). This is due to socioeconomic similarity and the fact that they share common cultural practices.

This study has both strengths and limitations. The strengths of this study include the use of a community-based methodology to identify the socioeconomic, demographic, and health-related aspects that determine enrolment decisions in CBHI. The results of this study also have the potential to significantly advance the body of knowledge in this area and offer insightful information to national stakeholders and policymakers. Importantly, the study was limited to a particular area in Ethiopia, which may limit the applicability of the conclusions. The respondents were asked to recall details about their choice to enroll in CBHI, which increases the likelihood of recall bias.

## 5 Conclusion

The results of this thorough study offer convincing evidence of the important aspects that strongly influence people's decisions to sign up for CBHI. The findings highlight the crucial role that formal education, perception, wealth index, community solidarity practices, trust in the CBHI scheme, quality of service, accessibility of necessary medications, and satisfaction with services play as key drivers of CBHI enrolment. Access to health insurance can be significantly enhanced by addressing and prioritizing advancements in education, perception, affordability, community involvement, trust, and service quality, which will ultimately result in better healthcare results overall. This study demonstrates the need for targeted interventions to improve community awareness, trust in the health system, and affordability of premiums. Strengthening community engagement, addressing service quality concerns, and improving accessibility can enhance enrolment in community-based health insurance and contribute to achieving universal health coverage in Ethiopia.

## Data Availability

The raw data supporting the findings of this study is available without undue reservation upon reasonable request from the corresponding author.
