# Supplementary material for: Determinants of the decision to enroll in community-based health insurance among households in the West Guji Zone, Oromia State, southern Ethiopia, in 2022
Source: Front Health Serv. 2025 May 15;5:1559578. doi: 10.3389/frhs.2025.1559578 (PMC12119507; doi:10.3389/frhs.2025.1559578)
Supplement: Supplementary file 1 [file Table1.pdf]

Supplementary Table 1: Socio-demographic characteristics for determinants of CBHI in the West Guji zone households, Oromia, Ethiopia, 2022

Attributes	Category	Cases (%)	Controls (%)
Household head	Male	240(69.6)	227(65.8)
	Female	105(30.4)	118(34.2)
Age	20-34	96(27.8)	81(23.5)
	35-49	213(61.7)	236(68.4)
	≥50	36(10.4)	28(8.1)
Marital status	In a married union	326(94.5)	301(87.2)
	Not in a married union	19(5.5)	44(12.8)
Educational status	No formal education	162(47)	136(39.4)
	Primary education	47(13.6)	64(18.6)
	Secondary education	108(31.3)	101(29.3)
	More than secondary education	28(8.1)	44(12.8)
Occupational status	Farmer	155(44.9)	124(35.9)
	Housewife	19(5.5)	36(10.4)
	Merchant	109(31.6)	112(32.5)
	Daily laborer	23(6.7)	21(6.1)
	Private employee	24(7)	30(8.7)
	Others	15(4.3)	22(6.4)
Family size of the households	Less than 5	121(35.1)	124(35.9)
	≥5	224(64.9)	221(64.1)
Children <18 years old	Yes	287(83.2)	259(75.1)
	No	58(16.8)	86(24.9)
Are there elderly > 65 years old?	Yes	215(62.3)	200(58)
	No	130(37.7)	145(42)
Religions of the households	Orthodox	52(15.1)	49(14.2)
	Muslim	43(12.5)	51(14.8)
	Protestant	173(50.1)	169(49)
	Wakefata	76(22)	76(22)
	Catholic	1(0.3)	
Participation in social solidarity practices	Yes	308(89.3)	238(69.0)
	No	37(10.7)	107(31.0)
Wealth quintile	Poor	127(36.8)	145(42)
	Medium	84(24.3)	104(30.1)
	Rich	134(38.8)	96(27.8)
Households' types of agriculture	Agrarian	84(24.3)	119(34.5)
	Pastoral	26(7.5)	43(12.5)
	Mixed	235(68.1)	183(53)
