# Supplementary material for: Determinants of the decision to enroll in community-based health insurance among households in the West Guji Zone, Oromia State, southern Ethiopia, in 2022
Source: Front Health Serv. 2025 May 15;5:1559578. doi: 10.3389/frhs.2025.1559578 (PMC12119507; doi:10.3389/frhs.2025.1559578)
Supplement: Supplementary file 2 [file Table2.pdf]

Supplementary Table 2: Perception of the households towards the CBHI among the West Guji zone households, Oromia, Ethiopia, 2022

Attributes	Cases (%)	Controls (%)
How satisfied are you with the adequacy of CBHI benefit packages in meeting the healthcare needs of your household?		
Very dissatisfied	8(2.3)	94(27.2)
Dissatisfied	16(4.6)	97(28.1)
Neutral	13(3.8)	49(14.2)
Satisfied	239(69.3)	66(19.1)
Completely satisfied	69(20)	39(11.3)
How trustworthy do you perceive the CBHI scheme to be?		
Not trustworthy at all	5(1.4)	64(18.6)
Slightly trustworthy	5(1.4)	110(31.9)
Neutral	10(2.9)	44(12.8)
Very trustworthy	145(42)	57(16.5)
Extremely trustworthy	180(52.2)	70(20.3)
How satisfied are you with the quality of health care services (waiting time, availability of drugs, diagnostics)?		
Very dissatisfied	6(1.7)	71(20.6)
Dissatisfied	5(1.4)	97(28.1)
Neutral	15(4.3)	48(13.9)
Satisfied	179 (51.9)	73(21.2)
Completely satisfied	140 (40.6)	56(16.2)
How satisfied are you with the diagnosis of your disease by the healthcare provider?		
Very dissatisfied	15(4.3)	77(22.3)
Dissatisfied	3(0.9)	86(24.9)
Neutral	11(3.2)	42(12.2)
Satisfied	125(36.2)	68(19.7)
Completely satisfied	191(55.4)	72(20.9)
Level of Perceptions of Households		
Negative	38(11)	243(70.4)
Positive	307(89)	102(29.6)
