# Supplementary material for: Determinants of the decision to enroll in community-based health insurance among households in the West Guji Zone, Oromia State, southern Ethiopia, in 2022
Source: Front Health Serv. 2025 May 15;5:1559578. doi: 10.3389/frhs.2025.1559578 (PMC12119507; doi:10.3389/frhs.2025.1559578)
Supplement: Supplementary file 3 [file Table3.pdf]

Supplementary Table 3: Medical and health-related characteristics of cases and controls, West Guji zone households, Oromia, Ethiopia, 2022

Attributes		Cases (%)	Control (%)
Is there a person with a non-communicable disease in your home?			
	Yes	128(37.1)	146(42.3)
	No	217(62.9)	199(57.7)
Is there an individual who frequently falls ill with a communicable disease?			
	Yes	28(8.1)	35(10.1)
	No	317(91.9)	310(89.9)
"Does the provision of service include prescribed drugs?"			
	Yes	260(75.4)	163(47.2)
	No	85(24.6)	182(52.8)
"Are you satisfied with the service that has been provided?"			
	Yes	260(75.4)	126(36.5)
	No	85(24.6)	219(63.5)
How long will it take to reach the nearest health centres or hospital?			
	<1hr	151(43.8)	118(34.2)
	1-2hr	117(33.9)	109(31.6)
	>2hr	77(22.3)	118(34.2)
How long it take to receive the service after you have paid for the treatment service?			
	<15min	77(22.3)	60(17.4)
	15-30	122(23.2)	118(34.2)
	31-45	111(32.2)	106(30.7)
	>46-60	31(9)	57(16.5)
	>60	4(1.2)	4(1.4)
