# Supplementary material for: Determinants of the decision to enroll in community-based health insurance among households in the West Guji Zone, Oromia State, southern Ethiopia, in 2022
Source: Front Health Serv. 2025 May 15;5:1559578. doi: 10.3389/frhs.2025.1559578 (PMC12119507; doi:10.3389/frhs.2025.1559578)
Supplement: Supplementary file 4 [file Table4.pdf]

Supplementary Table 4: Output of bivariable and multivariable logistic regression results of determinants of enrollment decision to CBHI among households of West Guji Zone, Oromia, Ethiopia, 2022

**Factors**	**Cases (%)**	**Controls (%)**	**COR (95% CI)**	**AOR (95% CI)**
**Respondents' educational status**				
No formal education	162(47)	136(39.4)	1.87(1.106, 3.167)	2.89(1.252, 6.648) *
Primary education	47(13.6)	64(18.6)	1.15(.630, 2.114)	1.37(.526, 3.560)
Secondary education	108(31.3)	101(29.3)	1.68(.973, 2.901)	1.69(.717, 3.984)
≥Tertiary education	28(8.1)	44(12.8)	1	1
**Do you participate in social solidarity practices like Equb and Edir?**				
Yes	308(89.3)	238(69.0)	3.74(2.483, 5.641)	4.27(2.352, 7.736) **
No	37(10.7)	107(31.0)	1	1
The wealth status of the respondents				
Poor	127(36.8)	145(42)	.63(.440, .894)	.31(.166, .569) **
Medium	84(24.3)	104(30.1)	.58(.392, .854)	.44(.226, .843) *
Rich	134(38.8)	96(27.8)	1	1
**Perception of the households toward CBHI**				
Negatives perception	38(11)	243(70.4)	.05(.035, .078)	0.07(.040, 0.112) **
Positive perception	307(89)	102(29.6)	1	1
Are prescribed drugs available during the provision of service				
Yes	260(75.4)	163(47.2)	3.42(2.471, 4.720)	1.83(1.102, 3.035) *
No	85(24.6)	182(52.8)	1	1
Have you been satisfied with the health facilities' service provision?				
Yes	260(75.4)	126(36.5)	5.32(3.827, 7.385)	3.21(1.937, 5.315) **
No	85(24.6)	219(63.5)	1	1
How do you rate the service as compared to payers?				
Good	233(67.5)	75(21.7)	7.49(5.326, 10.532)	2.21(1.324, 3.687) **
Poor	112(32.5)	270(78.3)	1	1
Have trust in CBHI scheme management/				
Yes	256(74.2)	92(26.7)	7.91(5.634, 11.105)	4.78(2.926, 7.816) **
No	89(25.8)	253(73.3)	1	1

NB: 1= Reference, *=significant at p<0.05, **=significant at p<0.001
